# Standing and Sitting Lumbopelvic-Hip Alignments and Mobilities Predict Proximal Junction Kyphosis and Hip Osteoarthritis Following Spinopelvic Fusion Surgery

**DOI:** 10.7759/cureus.91312

**Published:** 2025-08-30

**Authors:** Takuhei Kozaki, Takahiro Kozaki, Hiroshi Hashizume, Hiroshi Iwasaki, Shunji Tsutsui, Masanari Takami, Daisuke Nishiyama, Hiroshi Yamada

**Affiliations:** 1 Department of Orthopaedic Surgery, Saiseikai Wakayama Hospital, Wakayama, JPN; 2 Department of Orthopaedic Surgery, Wakayama Medical University, Wakayama, JPN

**Keywords:** adjacent joint disease, adjacent segment disease, adult spinal deformity, hip osteoarthritis, hip user, proximal junctional kyphosis, spine user, spinopelvic-hip mobility

## Abstract

Background: This study evaluated spinal alignments and spine-hip mobilities between sitting and standing positions in the development of proximal junctional kyphosis (PJK) and hip osteoarthritis (HOA) in adult spinal deformity (ASD) surgery.

Methods: This study included 90 patients (82 female, eight male; mean age=72.5 years) who underwent ASD surgery. Lumbo-pelvic parameters, including lumbar lordosis (LL), sacral slope (SS), and sacro-femoral angle (SFA), were measured in standing and sitting positions based on plain radiographs. The mobilities of each parameter were calculated between standing and sitting (ΔLL, ΔSS, ΔSFA). Proximal junctional angle (PJA) and Kellgren-Lawrence (KL) grade were compared between preoperative and postoperative values. The relationships between complications of ASD surgery and spinopelvic-hip alignments in standing and sitting positions were assessed.

Results: The cut-off value of ΔLL for predicting PJK was 20 degrees and of ΔSFA for predicting HOA was 70 degrees, based on receiver operating curve parameters. We defined patients with a ΔLL of more than 20 degrees as spine users and a ΔSFA of more than 70 degrees as hip users. About 22 (24.4%) patients had PJK, and 16 (17.8%) patients developed progression of HOA. Being a spine user and the presence of PJA at pre-operation were statistically significant predictive factors for PJK. Progression of HOA was significantly related to being a hip user and the KL grade at pre-operation.

Conclusion: This study is the first to demonstrate that lumbar and hip mobilities before spinopelvic fixation in ASD patients may predict PJK and HOA postoperatively, respectively.

## Introduction

Adult spinal deformity (ASD) is a spinal disorder that manifests in the aging population [1]. ASD is often associated with severe low back pain and can lead to a decrease in quality of life [2]. In patients who do not respond to conservative management, surgical intervention is one option to address ASD and improve quality of life [3]. However, surgical procedures for ASD remain challenging due to various perioperative and postoperative complications [4]. Among these concerns, proximal junctional kyphosis (PJK) is one of the most common complications following spinal fusion surgery [5]. PJK occurs as a result of symptomatic degeneration of spinal levels adjacent to a fused segment [6,7]. The development of PJK is problematic because it adversely affects functional outcome and can lead to instrumentation failure, neurologic deficit, upper instrumented vertebra (UIV) collapse, and acute subluxation [8]. In addition, “early-onset” hip osteoarthritis (HOA) may develop and progress after spinal fusion surgery [9]. In one study lasting more than two years, the prevalence of HOA was 10%, with pelvis fixation being a prominent risk factor [10]. Biomechanical studies have also shown that spinopelvic fusion surgery increases stress at the hip joint [11]. 

Due to the potential complications associated with PJK and HOA, it is important to reduce their occurrence and aim for optimal radiologic and clinical outcomes. Numerous other risk factors of PJK are mentioned in the literature, but the alignments of the spine and hip in the sitting posture have recently been proposed as key determinants for PJK [12,13]. Spinopelvic-hip movement patterns (i.e., greater hip flexion during daily activity) may lead to increased risk of anterior hip impingement [14]. Furthermore, patients with lumbar disc degeneration have decreased spinal mobility, leading to compensatory effects on greater hip motion, which induces anterior hip impingement [15]. Spinopelvic fusion increases the risk for anterior impingement in patients who undergo total hip arthroplasty (THA) [16,17]. 

Although several reports have proposed relationships between spinopelvic-hip movement patterns and complications in ASD surgery, these remain unclear. We suspect that patients with large spinal motion before fusion surgery may lose it after fusion surgery and compensate by increasing mobility at adjacent spinal segments, which might lead to PJK. We also suspect that patients with hypermobility of the hip joint before fusion surgery are at increased risk of HOA. Therefore, the aim of this study was to investigate whether preoperative spinopelvic-hip alignments during standing and sitting can predict the development of PJK and HOA after spinal fusion with pelvic fixation surgery.

## Materials and methods

Study design and population

**Figure 1:**
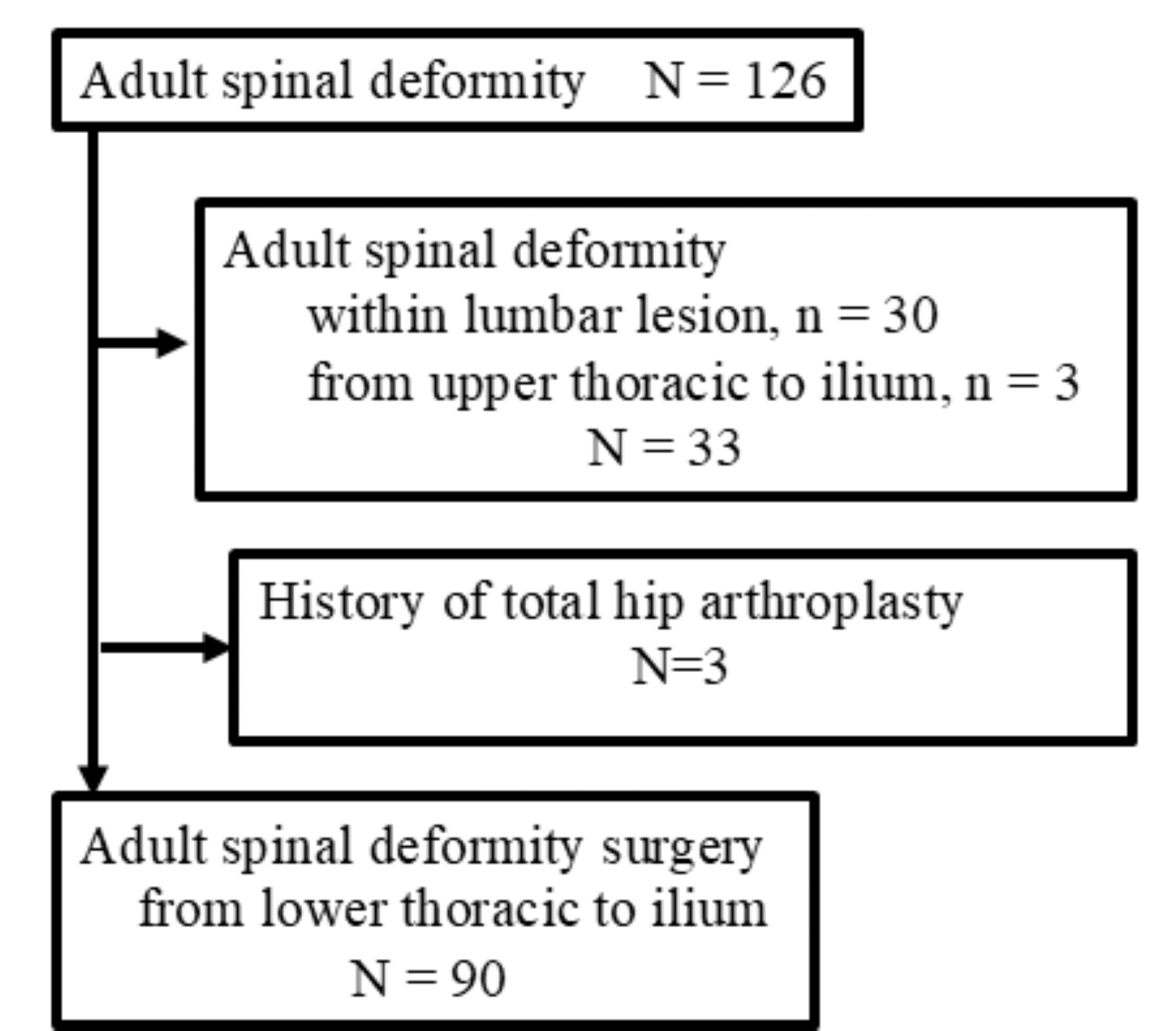
Flow diagram of the present study

This was a retrospective study with prospectively collected data of spine surgery patients at Wakayama Medical University Hospital, Wakayama, Japan. The study was approved by the institutional ethics board (reference no. 2511) and conducted according to the principles expressed in the Declaration of Helsinki. Informed consent was obtained from all the patients. A total of 126 patients who underwent ASD surgery from January 2019 to April 2021, with a minimum follow-up period of two years, were included in the study. The inclusion criteria consisted of adults with degenerative kyphoscoliosis who underwent instrumented spinal fusion surgery from the lower thoracic spine to the pelvis. The exclusion criteria were as follows: fusion within a lumbar lesion (n=30), fusion from the upper thoracic to the pelvis (n=3), and history of THA (n=3). Finally, 90 patients were included in the study (Figure 1).

Imaging measurements 

**Figure 2:**
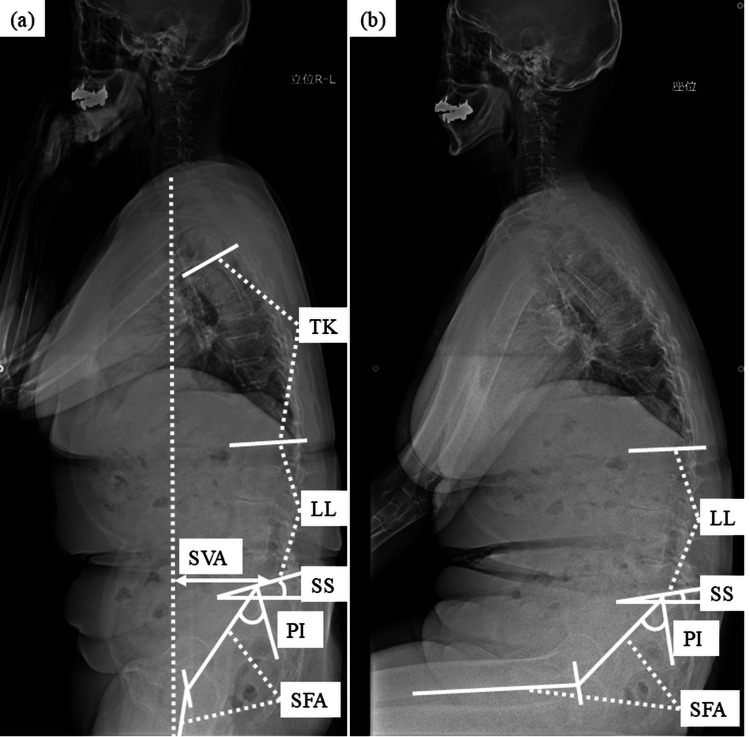
Whole-spine lateral radiographs in standing (a) and sitting (b) postures LL: Lumbar lordosis; SS: sacral slope; SFA: sacro-femoral angle; PI: pelvic incidence; TK: thoracic kyphosis; SVA: sagittal vertical axis

All patients had standing and sitting preoperative plain radiographs and standing plain radiographs at final follow-up. Postoperative measurements were performed two years after surgery in all patients. In cases requiring reoperation due to vertebral fracture, the measurements were obtained immediately prior to the reoperation. Sitting measurements were taken in a relaxed position to evaluate daily mobility, where the patient assumed their natural sitting posture on a chair [18]. Lumbar lordosis (LL), sacral slope (SS), and sacro-femoral angle (SFA) were also measured in the standing and sitting positions (Figures 2a-2b) [19,20].

**Figure 3:**
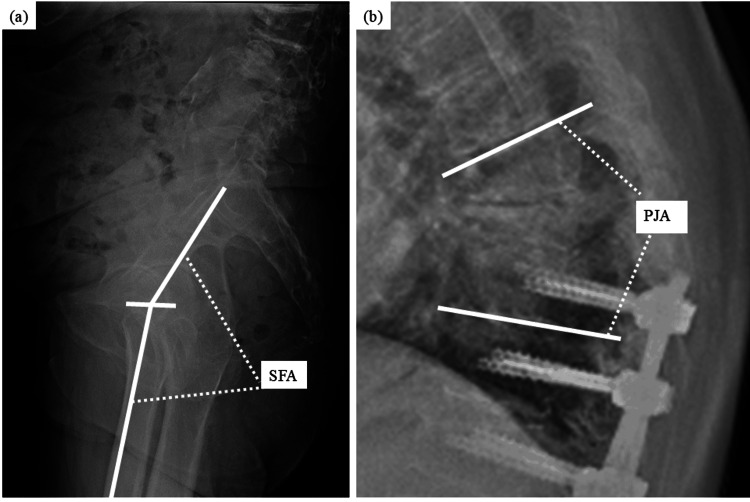
Lateral radiographs of the hip joint (a) and thoracic spine (b) SFA: sacro-femoral angle; PJA: proximal junctional angle

LL was defined as the angle between the upper superior endplate of L1 and the sacrum. SS was defined as the slope of the upper endplate of the sacrum from the horizontal line. SFA was defined as the angle between the line intersecting the center of the femoral head and the midpoint of the sacral plate and the line intersecting the center of the femoral head and the shaft of the femoral bone (Figure 3a). ΔLL was defined as the variation in LL when transitioning from standing to sitting positions before surgery, with ΔSS and ΔSFA defined in the same way. The sagittal vertical axis (SVA), pelvic incidence (PI), thoracic kyphosis (TK), and proximal junctional angle (PJA) were measured based on the standing radiograph. SVA was defined as the horizontal distance from the C7 plumb line to the posterior superior corner of the superior margin of the sacrum [20]. PI was defined as the angle between the perpendicular plane to the midpoint of the upper endplate of S1 and the line intersecting this point to the femoral axis [21]. TK was defined as the kyphotic angle between the upper endplate of T5 and lower endplate of T12 (kyphosis: +) [20]. PJA was defined as the angle between the inferior endplate of the UIV and the superior endplate of two levels above the UIV (kyphosis; +) (Figure 3b).

HOA was evaluated based on the Kellgren-Lawrence (KL) grading system in the standing X-ray [22]. KL grades are categorized as follows: KL grade 0 (KL0), no osteoarthritis; KL grade 1 (KL1), doubtful; KL grade 2 (KL2), mild; KL grade 3 (KL3), moderate; KL grade 4 (KL4), severe [22]. HOA was defined as the postoperative progression of the KL grade after the spinal fusion surgery. The reliability of continuous variables was assessed in terms of the intraclass correlation coefficient (ICC), while the reliability of categorical variables was evaluated using Cohen’s kappa coefficient. ICC values were interpreted as follows: <0.5, poor; 0.5-0.75, moderate; 0.75-0.9, good; >0.9, excellent. Kappa values were interpreted as follows: ≤0.20, slight agreement; 0.21-0.40, fair agreement; 0.41-0.60, moderate agreement; 0.61-0.80, substantial agreement; >0.80, almost perfect agreement. 

PJK Risk Analysis (analysis 1)

The cohort was divided into two groups: those with PJK (Group K) and those without PJK (Group NK). PJK was defined as a postoperative PJA increase of more than 20 degrees [23]. 

HOA Risk Analysis (Analysis 2)

The participants were divided into two groups: those with progression of HOA (Group H) and those with non-progression of HOA (Group NH). Progression of HOA was defined as the postoperative progression of the KL grade after the spinal fusion surgery. 

Statistical analyses

JMP Pro 14.1 (SAS Institute Inc., Cary, USA) was used for all statistical analyses. We compared data between the groups by using univariate analysis. Chi-square tests were used for categorical data, and Student’s t-test was used for continuous data. Receiver operating characteristic curves were used to determine the predictability of the evaluation in the occurrence of PJK and HOA and to calculate their cut-off values. Area under the curve (AUC) was used to evaluate the validity of the thresholds. A p-value of less than 0.05 was considered statistically significant. All numerical data were expressed as means ± standard deviations.

## Results

The mean age of the study participants was 72.5±5.7 years. Of the 90 patients, eight were male and 82 were female. The mean body mass index was 23.7±2.9 kg/m². The number of the fusion segments was 10.9±1.2. About 22 (24.4%) patients had PJK, and 16 (17.8%) patients had HOA (Table 1).

**Table 1:** Preoperative characteristics of the patients LL: lumbar lordosis; SS: sacral slope; SFA: sacro-femoral angle; SVA: sagittal vertical axis; TK: thoracic kyphosis; PI: pelvic incidence; PJA: proximal junctional angle

Characteristics	Values of all participants (n=90), mean±SD
Age (years)	72.5±5.7
Male, n (%)	8 (8.9%)
Female, n (%)	82 (91.1%)
Body mass index (kg/m²)	23.7±2.9
LL in standing position (°)	0.78±23.7
LL in sitting position (°)	-6.9±21.5
ΔLL (°)	11.6±10.3
SS in standing position (°)	11.4±13.9
SS in sitting position (°)	4.1±15.1
ΔSS (°)	10.7±8.2
SFA in standing position (°)	157.6±10.7
SFA in sitting position (°)	222.5±15.4
ΔSFA (°)	65.1±13.3
SVA in standing position (mm)	116.7±46.2
TK in standing position (kyphosis; +) (°)	22.8±14.9
PI in standing position (°)	49.4±11.6
PI-LL in standing position (°)	46.5±22.7
PJA in standing position (kyphosis; +) (°)	6.4±8.5
Number of fusion segments (segments)	10.9±1.2

The ICC values for interobserver (intraobserver) reliability for sitting LL, SS, and SFA were 0.81 (0.92), 0.85 (0.97), and 0.78 (0.91), respectively. The ICC values for interobserver (intraobserver) reliability for standing LL, SS, SFA, TK, SVA, PI, and PJA were 0.83 (0.95), 0.88 (0.98), 0.81 (0.93), 0.82 (0.89), 0.81 (0.88), 0.88 (0.90), and 0.83 (0.92), respectively, which were all excellent or good (Table 2).

**Table 2:** ICC values for interobserver and intraobserver reliability of each parameter LL: lumbar lordosis; SS: sacral slope; SFA: sacro-femoral angle; SVA: sagittal vertical axis; TK: thoracic kyphosis; PI: pelvic incidence; PJA: proximal junctional angle; ICC: intraclass correlation coefficient

Parameter	Interobserver ICC	Intraobserver ICC
LL in standing position (°)	0.83	0.95
LL in sitting position (°)	0.81	0.92
SS in standing position (°)	0.88	0.98
SS in sitting position (°)	0.85	0.97
SFA in standing position (°)	0.81	0.93
SFA in sitting position (°)	0.78	0.91
SVA in standing position (mm)	0.81	0.88
TK in standing position (°)	0.82	0.89
PI in standing position (°)	0.88	0.9
PJA in standing position (°)	0.83	0.92

**Figure 4:**
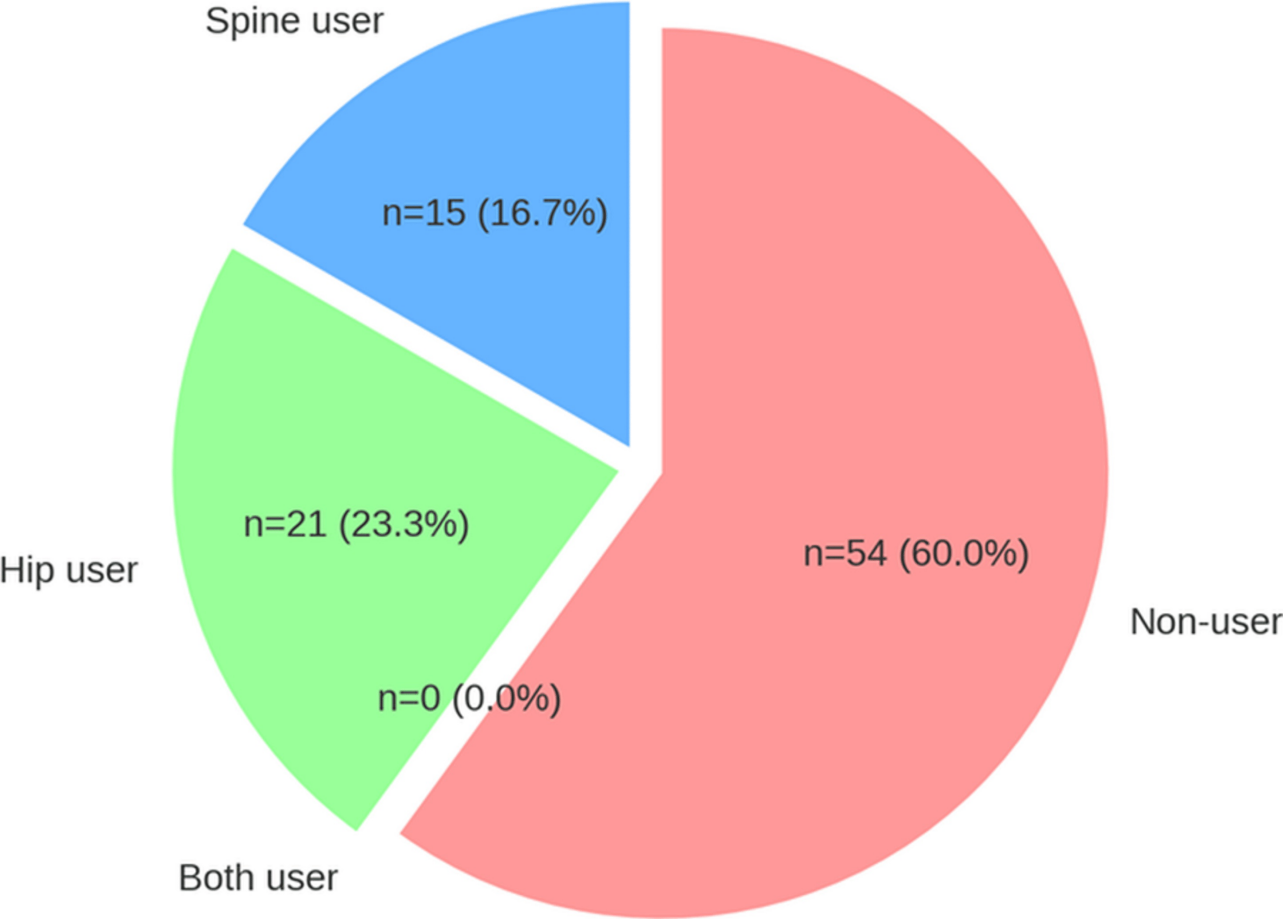
Distribution of user categories

Cohen’s kappa coefficients (k) for interobserver and intraobserver reliability in KL grading were 0.95 and 0.98, respectively, both of which indicated almost perfect agreement. The cut-off values of ΔLL for predicting PJK and ΔSFA for predicting HOA were 20 degrees (AUC=0.78, sensitivity=0.62, 1-specificity=0.05, Youden index=0.56) and 70 degrees (AUC=0.80, sensitivity=0.73, 1-specificity=0.13, Youden index=0.60), respectively. We defined patients with a ΔLL of more than 20 degrees as spine users and a ΔSFA of more than 70 degrees as hip users. About 15 (16.7%) patients were spine users, and 21 (23.3%) patients were hip users. No patient had a ΔLL of more than 20 degrees or a ΔSFA of more than 70 degrees (both user). The remaining 54 (60.0%) patients all had a ΔLL of less than 20 degrees and a ΔSFA of less than 70 degrees (non-user) (Figure 4).

PJK risk analysis (analysis 1)

On average, preoperative PJA was more lordotic in Group K (-0.4±7.2 degrees) than in Group NK (8.7±7.7 degrees) (p<0.0001), whereas postoperative PJA was more kyphotic in Group K (27.7±8.6 degrees) than in Group NK (14.3±7.1 degrees) (p<0.0001). Change in PJA was larger in Group K (28.1±7.6 degrees) than in Group NK (5.7±8.2 degrees) (p<0.0001). ΔLL was larger in Group K (16.5±9.7 degrees) than in Group NK (10.0±10.0 degrees) (p=0.0013). There were more spine users in Group K (n=10, 45.5%) than in Group NK (n=5, 7.4%) (p<0.0001). Postoperative TK was more kyphotic in Group K (46.4±24.8 degrees) than in Group NK (38.3±12.4 degrees) (p=0.0068), but preoperative TK was not significantly different between groups. Change in TK was larger in Group K (28.2±17.3 degrees) than in Group NK (14.4±12.9 degrees) (p<0.0001) (Table 3).

**Table 3:** Comparison between the PJK occurrence group (Group K) and the non-occurrence group (Group NK) Categorical variables were compared using the chi-square test. Continuous variables were analyzed using the Mann–Whitney U test. A p-value of <0.05 was considered statistically significant. LL: lumbar lordosis; SS: sacral slope; SFA: sacro-femoral angle; SVA: sagittal vertical axis; TK: thoracic kyphosis; PI: pelvic incidence; PJA: proximal junctional angle; PJK: proximal junctional kyphosis

Parameter	Group K (n=22), mean±SD	Group NK (n=68), mean±SD	p value
Age (years)	73.2±4.5	72.3±6.0	0.48
Male, n (%)	2 (9.1%)	6 (8.8%)	0.97
Female, n (%)	20 (90.9%)	62 (91.2%)	
Body mass index (kg/m²)	23.8±2.0	23.7±3.2	0.64
LL in standing position (°)	4.7±20.0	-0.5±24.7	0.30
LL in sitting position (°)	-7.2±24.7	-6.8±20.6	0.88
ΔLL (°)	16.5±9.7	10.0±10.0	0.0013
Spine user: yes, n (%)	10 (45.5%)	5 (7.4%)	<0.0001
Spine user: no, n (%)	12 (54.5%)	63 (92.6%)	
SS in standing position (°)	12.4±13.1	11.4±14.3	0.85
SS in sitting position (°)	5.1±15.1	3.7±15.2	0.76
ΔSS (°)	12.1±8.5	10.3±8.1	0.32
SFA in standing position (°)	161.8±13.4	156.3±9.4	0.093
SFA in sitting position (°)	222.6±12.3	222.4±16.4	0.66
ΔSFA (°)	61.9±16.3	66.2±12.1	0.76
Hip user: yes, n (%)	3 (13.6%)	18 (26.5%)	0.21
Hip user: no, n (%)	19 (86.4%)	50 (73.5%)	
SVA at pre-operation (mm)	109.0±60.1	119.1±40.9	0.33
SVA at post-operation (mm)	49.6±38.5	44.1±34.8	0.59
Correction of SVA (mm)	73.4±66.2	75.9±68.7	0.75
TK at pre-operation (kyphosis; +) (°)	19.2±13.9	23.9±15.1	0.32
TK at post-operation (kyphosis; +) (°)	46.4±24.8	38.3±12.4	0.0069
Change of TK (°)	28.2±17.3	14.4±12.9	0.0001
PI at pre-operation (°)	48.9±8.1	49.5±12.5	0.82
PI at post-operation (°)	48.6±7.9	50.1±10.6	0.81
Change of PI (°)	-0.2±5.3	0.6±5.1	0.29
PI-LL at pre-operation (°)	44.4±16.3	49.3±22.6	0.49
PI-LL at post-operation (°)	10.4±7.8	11.8±14.0	0.81
Change of PI-LL (°)	34.0±15.7	37.5±20.6	0.37
PJA at pre-operation (kyphosis; +) (°)	-0.4±7.2	8.7±7.7	<0.0001
PJA at post-operation (kyphosis; +) (°)	27.7±8.6	14.3±7.1	<0.0001
Change of PJA (°)	28.1±7.6	5.7±8.2	<0.0001
Number of fusion segments (segments)	10.7±0.84	11.0±1.3	0.25

HOA risk analysis (analysis 2)

ΔSFA was greater in Group H (72.6±16.7 degrees) than in Group NH (63.5±11.9 degrees) (p<0.0001). There were more hip users in Group H (n=11, 68.8%) than in Group NH (n=10, 13.5%) (p<0.0001). Preoperative KL grade was more progressed in Group H (KL0: 8, KL1: 3, KL2: 2, KL3: 3, KL4: 0) than in Group NH (KL0: 73, KL1: 0, KL2: 1, KL3: 0, KL4: 0) (p<0.0001). Postoperative KL grade was more progressed in Group H (KL0: 0, KL1: 5, KL2: 2, KL3: 6, KL4: 3) than in Group NH (KL0: 73, KL1: 0, KL2: 1, KL3: 0, KL4: 0) (p<0.0001) (Table 4).

**Table 4:** Comparison between the HOA progression group (Group H) and the non-progression group (Group NH) Categorical variables were compared using the chi-square test. Continuous variables were analyzed using the Mann-Whitney U test. A p-value of <0.05 was considered statistically significant. LL: lumbar lordosis; SS: sacral slope; SFA: sacro-femoral angle; SVA: sagittal vertical axis; TK: thoracic kyphosis; PI: pelvic incidence; KL: Kellgren-Lawrence; HOA: hip osteoarthritis

Parameter	Group H (n=16), mean±SD	Group NH (n=74), mean±SD	p value
Age (years)	71.9±6.8	72.8±5.4	0.42
Male, n (%)	0 (0.0%)	8 (10.8%)	0.17
Female, n (%)	16 (100.0%)	66 (89.2%)	
Body mass index (kg/m²)	23.8±2.1	23.8±3.1	0.72
LL in standing position (°)	1.8±18.4	0.7±24.4	0.78
LL in sitting position (°)	-8.0±15.3	-6.7±22.1	0.94
ΔLL (°)	11.9±11.8	11.7±10.1	0.76
Spine user: yes, n (%)	2 (12.5%)	13 (17.6%)	0.62
Spine user: no, n (%)	14 (87.5%)	61 (82.4%)	
SS in standing position (°)	12.2±12.1	11.3±14.2	0.87
SS in sitting position (°)	4.6±11.8	3.9±15.5	0.90
ΔSS (°)	9.0±6.6	11.1±8.4	0.35
SFA in standing position (°)	155.4±7.0	157.9±11.1	0.17
SFA in sitting position (°)	229.5±12.4	221.6±15.7	0.022
ΔSFA (°)	74.1±15.8	64.1±12.2	<0.0001
Hip user: yes, n (%)	11 (68.8%)	10 (13.5%)	<0.0001
Hip user: no, n (%)	5 (31.2%)	64 (86.5%)	
SVA at pre-operation (mm)	107.5±49.9	117.3±46.2	0.32
SVA at post-operation (mm)	47.6±35.0	46.8±37.7	0.97
Correction of SVA (mm)	67.8±51.0	77.9±71.0	0.97
TK at pre-operation (kyphosis; +) (°)	19.0±12.2	23.8±14.8	0.23
TK at post-operation (kyphosis; +) (°)	39.6±11.5	40.3±17.4	0.63
Change of TK (°)	22.7±11.7	16.5±15.7	0.24
PI at pre-operation (°)	48.9±12.7	49.4±11.5	0.80
PI at post-operation (°)	48.5±9.4	50.1±10.2	0.82
Change of PI (°)	0.3±4.6	-0.03±5.4	0.58
PI-LL at pre-operation (°)	49.5±12.7	48.0±22.6	0.91
PI-LL at post-operation (°)	12.2±9.0	11.2±13.5	0.51
Change of PI-LL (°)	37.3±12.6	36.8±20.7	0.93
KL grade at pre-operation			<0.0001
KL grade 0	8 (50.0%)	73 (98.6%)	
KL grade 1	3 (18.75%)	0 (0.0%)	
KL grade 2	2 (12.5%)	1 (1.4%)	
KL grade 3	3 (18.75%)	0 (0.0%)	
KL grade 4	0 (0.0%)	0 (0.0%)	
KL grade at post-operation			<0.0001
KL grade 0	0 (0.0%)	73 (98.6%)	
KL grade 1	5 (31.3%)	0 (0.0%)	
KL grade 2	2 (12.5%)	1 (1.4%)	
KL grade 3	6 (37.5%)	0 (0.0%)	
KL grade 4	3 (18.7%)	0 (0.0%)	
Number of fusion segments (segments)	11.8±2.0	10.9±1.0	0.090

**Figure 5:**
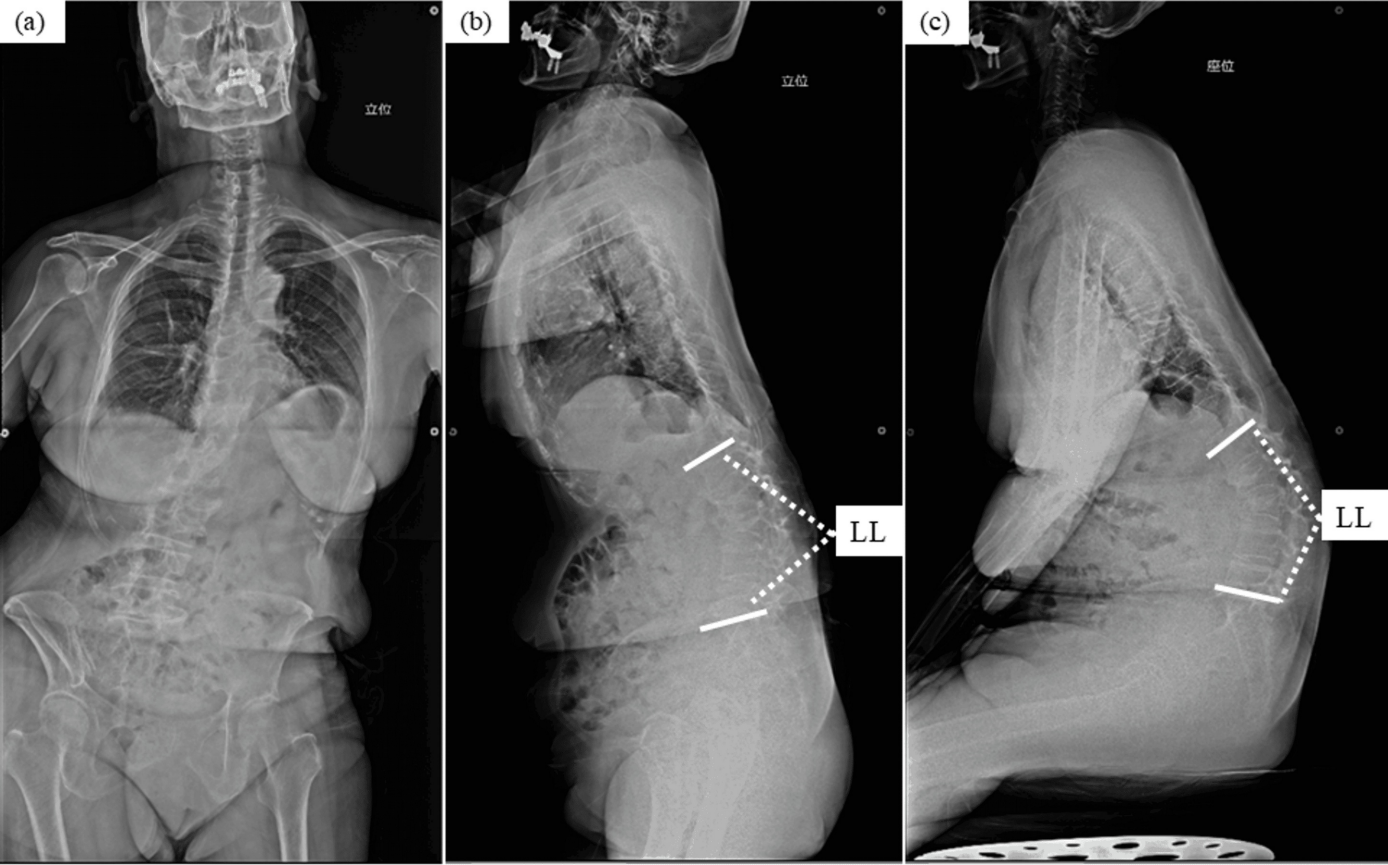
Case 1 - 70-year-old female patient The preoperative X-rays showed that LL was -19 degrees, SS was 7 degrees, and SFA was 176 degrees in the standing posture (a, b), and LL was -45 degrees, SS was -9 degrees, and SFA was 216 degrees in the sitting posture (c). Additionally, ΔLL was 26 degrees, ΔSS was 16 degrees, and ΔSFA was 40 degrees. She was a spine user. LL: lumbar lordosis; SS: sacral slope; SFA: sacro-femoral angle

**Figure 6:**
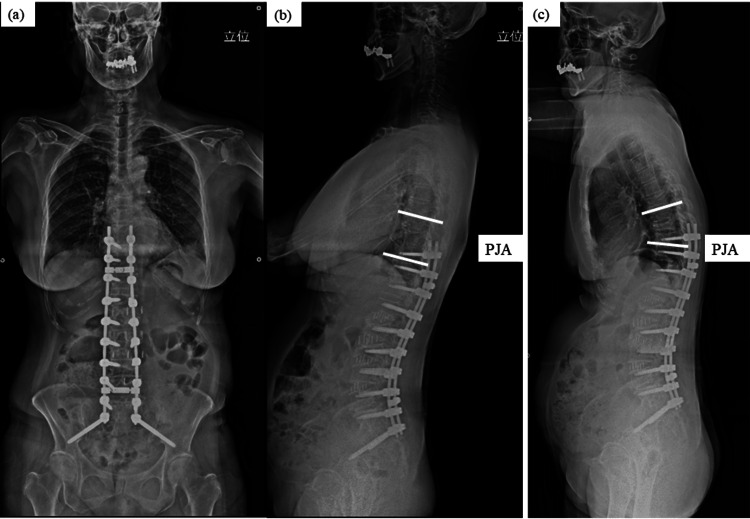
Case 1 - 70-year-old female patient She underwent spinopelvic fixation surgery (a, b) and experienced a vertebral fracture three months later. Six months later, she developed a kyphotic posture. The proximal junctional angle was 2 degrees one month after surgery (a), and it became 23 degrees one year after surgery (c).

**Figure 7:**
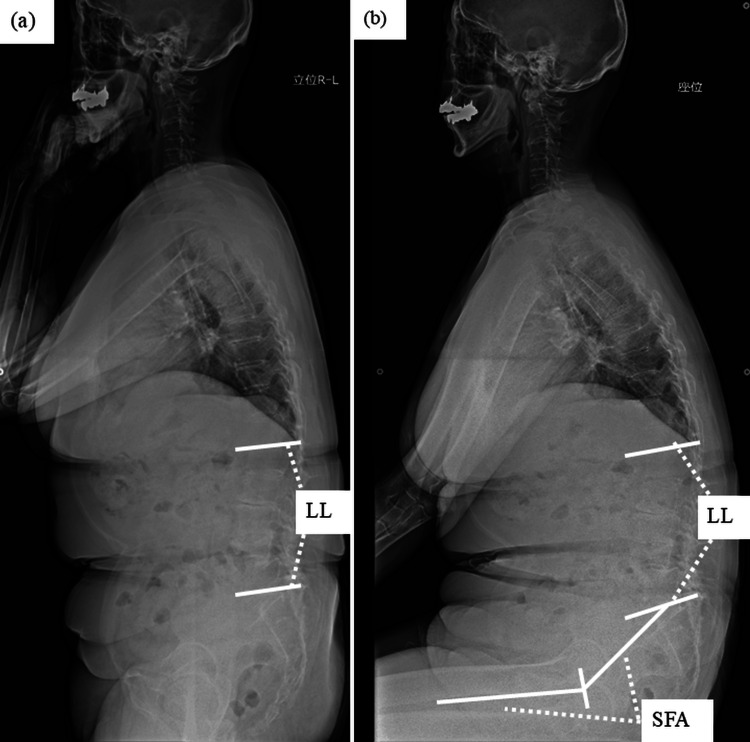
Case 2 - 70-year-old female patient The preoperative X-rays showed that LL was 6 degrees, SS was 10 degrees, and SFA was 155 degrees in the standing posture (a), and LL was 4 degrees, SS was 9 degrees, and SFA was 227 degrees in the sitting posture (b). Additionally, ΔLL was 2 degrees, ΔSS was 1 degree, and ΔSFA was 72 degrees. LL: lumbar lordosis; SS: sacral slope; SFA: sacro-femoral angle

**Figure 8:**
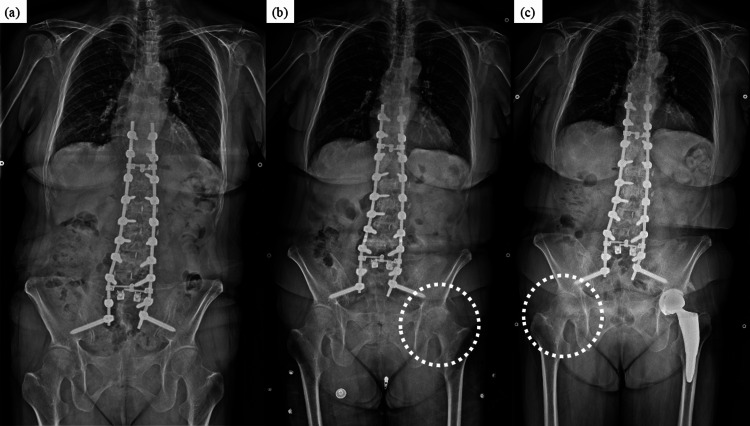
Case 2 - 70-year-old female patient Whole-spine anteroposterior X-rays in the standing posture at 2.5 years (a), 3.5 years (b), and five years (c) after surgery. No problems were observed at 2.5 years (a), but hip osteoarthritis on the left side had progressed by 3.5 years (b) and on the right side by five years (c).

Representative cases of a spine user and a hip user are shown in Figures 5-6 and Figures 7-8, respectively.

## Discussion

We investigated the effects of spinopelvic-hip alignments and mobilities during standing and sitting on PJK and HOA. Being a spine user and preoperative PJA were predictive factors for PJK. In addition, HOA was associated with being a hip user and preoperative KL grade.

Adjacent segment disease refers to radiographic degeneration and/or clinical deterioration at spinal levels adjacent to a fusion. Its proposed mechanism involves load transmission from the fused spine to the adjacent segments or joints, resulting in increased stresses and motion [24]. Its pathology is characterized by increased segmental motion and elevated pressures adjacent to the fused segments [25]. In addition, larger motion and stress are transferred to the adjacent segments as the number of fusion segments increases [25]. In this regard, we suspected that spine users at pre-operation may lose more motion and likely compensate at the residual unfused spinal segments, which might lead to a higher incidence of PJK. Similarly, hip users at pre-operation are likely to compensate for the loss of spinal mobility by increasing mobility at the hip joint instead of hypermobility of the adjacent spine. The results of this study seem to support these hypotheses.

Recently, it was reported that there is a significant difference in sagittal alignment between the standing and sitting postures [26]. In general, humans spend almost half of their waking time in a sitting posture. Other studies suggested that assuming a kyphotic posture during sitting was a predictive factor for PJK [12,13]. However, it is still unclear whether the dynamic motion between standing and sitting is related to complications in ASD surgery. In a previous study evaluating lumbar mobility differences between healthy individuals and those with lumbar disc degeneration, ΔLL was 36.5 degrees in the healthy group and decreased to 20.6 degrees in the degeneration group [15]. The mean ΔLL was 11.1 degrees, which was attributed to more severe degenerative change in ASD patients than in past studies [27]. Furthermore, the mobility of the hip joint was 55.4 degrees in the healthy group and 62.3 degrees in the lumbar disc degenerative group [15].

In the current study, the mean ΔSFA was 65.0 degrees. In ASD patients, larger compensatory mechanisms appeared to be necessary in the presence of more severe spinal degeneration compared to less degenerated cases. Being a spine user was significantly related to PJK after spinopelvic fixation surgery, whereas hip user status was associated with HOA progression following spinopelvic fixation surgery. Although the exact pathologies remain unclear, we believe that spine users may try to compensate for their loss of spinal mobility by increased use of the adjacent unfused spine, whereas hip users compensate through hypermobility of the hip joint.

More lordotic preoperative PJA was significantly associated with the development of PJK, which was thought to be related to lumbar kyphosis and more lordotic posture at lower thoracic vertebrae before surgery. In such cases, the kyphotic apex appeared to be located higher than the UIV, and compensatory kyphotic changes might occur in the adjacent upper segments following fixation, in an attempt to preserve sagittal alignment [12]. In retrospect, selecting a higher UIV might have been more appropriate to prevent the development of PJK in certain patients. On the other hand, progression of KL grade at pre-operation has been reported as a risk factor for HOA after spinopelvic fusion surgery [9,10]. Therefore, the spine surgeon should pay attention to the hip pathology before surgery.

Our study has several limitations. First, our sample size was small. Although our sample size was relatively small and insufficient to establish a new disease concept, we observed that ΔLL and ΔSFA were significantly correlated with PJK and HOA after spinal fusion surgery. These results suggest that assessing lumbopelvic motion could be a promising predictive factor for the development of PJK and HOA. Secondly, selection bias may have been present in our study. Specifically, our sample consisted of an older population compared to those in previous studies, and the majority of participants were female. These factors may have contributed to the lower flexibility of the spine and hip joint observed in our cohort. [15]. This tendency to avoid long spinopelvic fusion in younger patients is aimed at reducing the risk of developing HOA [28,29]. This study established definitions for spine users and hip users, but these definitions were limited to ASD subjects, and additional epidemiological studies are necessary. Thirdly, this study included only Japanese subjects, who have a lower prevalence of HOA than Western subjects [30]. The prevalence of HOA after spinal fusion surgery might be higher in Western subjects than in Japanese subjects. Finally, we did not evaluate alignments during standing and sitting at post-operation. We took relevant X-rays two years after fusion surgery, but we could not evaluate the ΔLL and ΔSFA equally. Although increased hip joint mobility was expected after fusion surgery, patients who developed HOA exhibited reduced mobility due to the progression of HOA. In the future, we should take the X-rays much earlier, such as six months after surgery.

## Conclusions

This study investigated the relationships between a spine user and hip user and postoperative PJK and HOA, respectively. Patients undergoing multilevel fusion surgery with pelvic fixation should be appropriately informed of these risks. Further studies with longer follow-up should be done to assess the true incidence of PJK and HOA over time and formulate preventive strategies. 
